# Bisphenol-A exposure and risk of breast and prostate cancer in the Spanish European Prospective Investigation into Cancer and Nutrition study

**DOI:** 10.1186/s12940-021-00779-y

**Published:** 2021-08-16

**Authors:** Elena Salamanca-Fernández, Miguel Rodríguez-Barranco, Pilar Amiano, Josu Delfrade, Maria Dolores Chirlaque, Sandra Colorado, Marcela Guevara, Ana Jimenez, Juan Pedro Arrebola, Fernando Vela, Nicolás Olea, Antonio Agudo, Maria-José Sánchez

**Affiliations:** 1 Andalusian School of Public Health (EASP), Campus Universitario de Cartuja, C/Cuesta del Observatorio 4, 18080 Granada, Spain; 2 Instituto de Investigación Biosanitaria ibs. GRANADA, Granada, Spain; 3 CIBER de Epidemiología y Salud Pública (CIBERESP), Madrid, Spain; 4 Public Health Division of Gipuzkoa, BioDonostia Research Institute, Donostia-San Sebastian, Spain; 5 Navarra Public Health Institute, Pamplona, Spain; 6 Navarra Institute for Health Research (IdiSNA), Pamplona, Spain; 7 Department of Epidemiology, Murcia Regional Health Council, IMIB-Arrixaca, Murcia, Spain; 8 Department of Health and Sciences, University of Murcia, Murcia, Spain; 9 Research Group on Demography and Health, National Faculty of Public Health, University of Antioquia, Medellín, Colombia; 10 Department of Preventive Medicine and Public Health, University of Granada, Granada, Spain; 11 Department of Radiology, University of Granada, Granada, Spain; 12 Unit of Nutrition and Cancer, Catalan Institute of Oncology - ICO, Nutrition and Cancer Group, Bellvitge Biomedical Research Institute - IDIBELL, L’Hospitalet de Llobregat, 08908 Barcelona, Spain

**Keywords:** Breast cancer, Prostate cancer, Case-cohort, Bisphenol a, Environmental pollutants

## Abstract

**Background:**

Bisphenol A (BPA) is an endocrine disruptor that it is present in numerous products of daily use. The aim of this study was to assess the potential association of serum BPA concentrations and the risk of incident breast and prostate cancer in a sub-cohort of the Spanish European Prospective Investigation into Cancer and Nutrition (EPIC).

**Methods:**

We designed a case-cohort study within the EPIC-Spain cohort. Study population consisted on 4812 participants from 4 EPIC-Spain centers (547 breast cancer cases, 575 prostate cancer cases and 3690 sub-cohort participants). BPA exposure was assessed by means of chemical analyses of serum samples collected at recruitment. Borgan II weighted Cox regression was used to estimate hazard ratios.

**Results:**

Median follow-up time in our study was 16.9 years. BPA geometric mean serum values of cases and sub-cohort were 1.12 ng/ml vs 1.10 ng/ml respectively for breast cancer and 1.33 ng/ml vs 1.29 ng/ml respectively for prostate cancer. When categorizing BPA into tertiles, a 40% increase in risk of prostate cancer for tertile 1 (*p* = 0.022), 37% increase for tertile 2 (*p* = 0.034) and 31% increase for tertile 3 (*p* = 0.072) was observed with respect to values bellow the limit of detection. No significant association was observed between BPA levels and breast cancer risk.

**Conclusions:**

We found a similar percentage of detection of BPA among cases and sub-cohort from our population, and no association with breast cancer risk was observed. However, we found a higher risk of prostate cancer for the increase in serum BPA levels. Further investigation is needed to understand the influence of BPA in prostate cancer risk.

**Supplementary Information:**

The online version contains supplementary material available at 10.1186/s12940-021-00779-y.

## Background

Cancer incidence is increasing worldwide with 18.1 million new cancer cases and 9.6 million cancer deaths in 2018 [1]. In Europe, breast and prostate cancers lead cancer incidence as they account for 13.5 and 12.6% of the newly diagnosed cases in 2018 respectively [2]. The same is observed in Spain, with 32,536 incident breast cancer cases and 34,394 prostate cancer cases in 2019 [3]. Some risk factors of these hormone dependent cancers are related to lifestyle: diet, smoking habit, weight, alcohol consumption and physical activity [4, 5]. However, owing to the hormonal dependence of these tumors, some environmental pollutants have the potential to act as carcinogens. In this regard, Bisphenol A (BPA) is considered an endocrine disruptor (ED) first developed in the 1890s. BPA is widely produced for the manufacture of polysulfones and polycarbonate plastic, polymers and epoxy resin, and thermal paper and it is one of the highest volume chemicals produced worldwide with 372,000 t produced in 2012 [6]. Therefore its presence is considered to be ubiquitous in the environment and human exposure is continuous [7, 8]. BPA has been detected in the urine (> 0.4 ng/ml) [9–12] of nearly 90% of adults and children as well as in the serum of general population, pregnant women, placenta, breast milk and amniotic fluid [13–18]. Humans are exposed to BPA through several routes: food (oral), occupation (inhalation) and contact materials, plastic type and medical devices (dermal) [7, 19]. However, the main exposure route of BPA is through diet, as many food packaging like tins, cans, plastic boxing etc. have BPA in their composition and it migrates to the food [7, 20–24].

BPA is considered a non-persistent chemical, i.e., as it is degraded in the organism and there is evidence that BPA acts as an endocrine disruptor with estrogenic effects in the rodent mammary gland [25–27]. Moreover, prenatal BPA exposure in rats induces preneoplastic lesions in the mammary [28]. Thus, studies conducted in vitro have shown that the exposure of the human breast cancer cell line to BPA increased its proliferation and caused increased oxidative stress [29]. In this regard, in vitro and animal studies have shown that BPA induces the proliferation of the androgen-sensitive human prostate cancer cells and increases epididymis weight [14, 30]. However, although BPA has been linked to hormone dependent cancer risk in animals its evidence in human is scare [31–33]. However, the number of epidemiological studies addressing this issue is growing [34–39]. Tough some of them have showed certain suffers from limitations the samples were collected after breast cancer diagnosis or have the limitation characteristic of retrospective case–control studies. In this study we have sought to avoid these limitations in our sample design and sampling.

The present study aims to assess the potential association of serum BPA concentrations and the risk of incident breast and prostate cancer in a sub-cohort of the Spanish European Prospective Investigation into Cancer and Nutrition (EPIC).

## Methods

### Study design

We designed a case-cohort study within the EPIC-Spain cohort. EPIC is a prospective multi-centric cohort study planned to investigate the relationship between diet, lifestyles and cancer. It involves 23 research centers in 10 European countries, including five Spanish centers: Asturias, Granada, Murcia, Navarra and Gipuzkoa [40]. Study participants reported information about dietary, lifestyle, reproductive and anthropometric factors at baseline.

### Study population

The EPIC-Spain included 41,446 participants (62% women) aged 29–69 years enrolled between 1992 and 1996 in five provinces of Spain. Participants were recruited mostly among blood donors (about 60%) and the study population covered a broad range of socioeconomic and educational levels. Furthermore, they signed an informed consent and the study was approved by Ethics Committee of the Bellvitge Hospital (Barcelona). EPIC study populations and data collection were explained elsewhere [41].

**Fig. 1:**
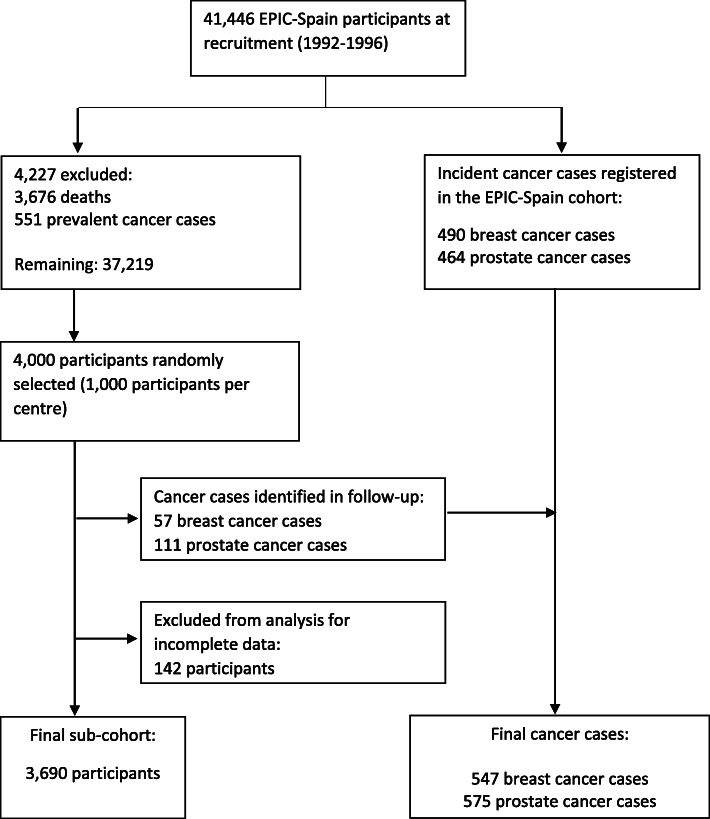
Flow chart: Case-cohort design of the study and the number of participants included in the analysis

Study population in the present study consisted of 3690 sub-cohort participants, 547 breast cancer cases and 575 prostate cancer cases from four EPIC-Spain centers (Gipuzkoa, Granada, Murcia and Navarra) with available data on BPA exposure. Participants selected for the sub-cohort included, by design, an overlap of 57 breast cancer cases and 111 prostate cancer cases (Fig. 1).

The sub-cohort was selected among participants registered as alive in the EPIC cohort until 30/12/2013 using stratified random sampling by sex (50% men and 50% women) and age (quintiles), excluding persons with cancer at recruitment. In our sub-cohort, 79.6% of the participants provided a fasting blood sample at recruitment and 85.6% of them were extracted between 6 am and 11 am. However, we can generalize and assume most of our samples were taken during the mornings and in fasting conditions.

Follow-up time began at EPIC recruitment and cancer cases were defined as participants with a diagnosis of breast or prostate cancer (i.e. ICD10 codes C50 and C61 respectively) during the study period. Incident cancer cases are identified by linkage with the Population Cancer Registries. Incidence date was determined by the date of cancer diagnosis and prevalent cases were excluded (participants with a cancer diagnosis prior to recruitment). Dates of end of follow-up for cases identification were: 31/12/2012 for Granada, 31/12/2013 for Murcia, 31/12/2011 for Navarra and 30/12/2013 for Gipuzkoa. For sub-cohort participants, the end of follow-up date was the lowest among date of diagnosis, date of death, date of loss of follow-up or the center-specific end-of-follow-up, whatever happened first.

Vital status and date of death of participants in our sub-cohort was determined by linking EPIC data base with the National and regional Registries, IND (death national index), INE (statistical national institute) and regional mortality register until 30/12/2013 (maximum date of identification of incident cases).

### Covariate assessment

Information on lifestyle and other health-related factors was obtained by an interviewer-administered questionnaire at baseline. All interviewers had received appropriate training for this task.

Measurements of height, weight, and hip and waist circumferences were taken at recruitment using standardised procedures [41]. The questionnaire included items on educational level, history of previous illnesses, history of tobacco use, alcohol consumption, physical activity, and reproductive history [41]. The participants were classified into three categories by body mass index (BMI): < 25 kg/m^2^, 25- < 30 kg/m^2^, ≥30 kg/m^2^. Educational level was classified according to five categories: none, primary school, secondary school, technical or vocational training and university degree. Smoking status was summarised in three categories: never smoked, former smoker and current smoker. Alcohol consumption at recruitment in grams per day was categorized as no drinker (0 g/day), drinker (≤30 g/d in men and ≤ 20 g/d in women) and heavy drinker (> 30 g/d in men and > 20 g/d in women). Information on the domains of physical activity was compiled taking seasonal variation into account. A simple four-level physical activity index (low, medium, high, very high) was derived and validated by combining recreational and household activity [42]. Reproductive history was additionally measured for women, including menopausal status at recruitment, number of pregnancies, breastfeeding (yes/no regardless of the duration), and use of oral contraceptive or hormone replacement therapy sometime in life.

### Sample collection and chemical analyses

Blood samples were drawn from each participant at recruitment, which were subsequently centrifuged, and aliquots of plasma, serum, red blood cells and buffy coat in 0.5 mL straws were stored in liquid nitrogen (− 196 °C).

BPA levels were quantified in serum samples using two of 0.5 mL straws, in an adaptation of a previously-validated methodology [43]. In brief, BPA was analysed by dispersive liquid–liquid micro-extraction (DLLME) and ultra-high performance liquid chromatography with tandem mass spectrometry detection (UHPLC-MS/MS). Samples were thawed completely at room temperature, centrifuged at 2600 g for 10 min and 0.75 mL was extracted for analysis. In order to determine total BPA (free plus conjugated) in serum, each sample was spiked with 50 μL of enzyme solution (β-glucuronidase/sulphatase) and incubated at 37 °C for 24 h. The treated serum was placed in a 15 mL screw-cap glass tube and spiked with 30 μL of the surrogate standard solution (1.25 mg/L of BPA-d16). The serum was then diluted to 10.0 mL with 5% NaCl aqueous solution (w/v) and the pH was adjusted to 2.0. Next, 0.75 mL of acetone and 0.75 mL of trichloromethane were mixed and injected rapidly into the aqueous sample with a syringe. After manual shaking, centrifugation and evaporation of the extract, the residue was dissolved with 100 μL of a mixture consisting of water (0.1% ammonia)/acetonitrile (0.1% ammonia), 70:30 (v/v), and finally 10 μL was injected into the LC system. Limit of detection (LOD) was 0.2 ng/ml. Values below LOD were assigned the LOD divided by the square root of 2.

Chemical analyses were performed at Centro de Excelencia en Investigación de Medicamentos Innovadores en Andalucía MEDINA (https://www.medinadiscovery.com/, which has been assessed and certified for the standards of ISO 9001:2015, and routinely performs internal and external quality control analyses. In addition, the present analyses are encompassed in the activities of our research group within the The Human Biomonitoring Initiative (HBM4EU, https://www.hbm4eu.eu/).

### Statistical analysis

Geometric means and 95% confidence intervals of the BPA levels (in ng/ml) were calculated for cases and sub-cohort and according to center, sex, age group, educational level, body mass index, physical activity and alcohol consumption and smoker status. Statistical differences were assessed through the Mann-Whitney or Kruskall-Wallis tests.

Time to cancer event were modelled by means of Borgan II weighted Cox proportional hazard models [44], stratified by center. Robust standard errors were used as recommended in such case-cohort design [45]. Hazard ratios and 95% confidence intervals were derived from these Cox models. BPA levels acted as principal independent variable, and were treated as continuous variable, transformed by base 2 logarithm to smooth their strong asymmetric distribution and categorized into tertiles to assess non-linear relationship. Multivariate Cox-regression models were always stratified by center and age group, and constructed using three strategies: A) linear BPA as independent variable; B) log2-transformed BPA as independent variable; C) categorized BPA as independent variable (considering a category with values <LOD as a reference and distributing the rest of the detectable values into terciles). The last two models allowed us to evaluate the non-linear relationship, which was also verified by the analysis of the martingale-based residuals in model A. All models were adjusted by age, education level, BMI, physical activity, smoking status, and alcohol consumption. For women, models were also adjusted by menopause, number of pregnancies, breastfeeding, oral contraceptives and hormone replacement therapy (HRT). An interaction term between BMI and menopause status was additionally included, since the join effect of these two factors can act as a modifier of breast cancer risk. The confounders were selected based on the evidence on factors potentially associated with the risk of the studied cancers among the variables available in the EPIC cohort. Statistical analysis was conducted with Stata v14 (﻿Stata Statistical Software: Release 14. College Station, TX: StataCorp LP).

## Results

In our study, median follow-up time was 17 years. We had 547 cases of breast cancer and 575 prostate cancer cases and 3690 sub-cohort participants (1918 women and 1772 men). Table 1 shows the main characteristics of cancer patients and sub-cohort participants of the study.

**Table 1:** Characteristics at recruitment of EPIC cancer cases and sub-cohort participants

		Breast Cancer			Prostate Cancer		
	Total	Cases	Sub-cohort	*p	Cases	Sub-cohort	*p
N (%)	4812 (100)	547 (22.1)	1918 (77.8)		575 (24.5)	1772 (75.5)	
**Center**				0.035			< 0.001
Gipuzkoa	1.239 (25.7)	123 (22.4)	467 (24.3)		225 (39.1)	424 (23.9)	
Granada	1159 (24.1)	116 (21.2)	501 (26.1)		63 (10.9)	479 (27.0)	
Murcia	1177 (24.4)	157 (28.7)	477 (24.8)		102 (17.7)	441 (24.8)	
Navarra	1237 (25.7)	151 (27.6)	473 (24.6)		185 (32.1)	428 (24.1)	
**Sex**				–	575 (24.5)	1772 (75.5)	–
Male	2347 (48.7)	–	–				
Female	2465 (51.2)	547 (22.2)	1918 (77.8)		–	–	
**Age**				< 0.001			< 0.001
< 45	936 (19.4)	174 (31.8)	367 (19.1)		34 (5.9)	361 (20.3)	
45–49	824 (17.1)	125 (22.8)	309 (16.1)		101 (17.5)	289 (16.3)	
50–54	994 (20.6)	96 (17.5)	399 (20.8)		120 (20.8)	379 (21.4)	
55–59	948 (19.7)	82 (14.9)	372 (19.4)		168 (29.2)	326 (18.4)	
60+	1110 (23.1)	70 (12.8)	471 (24.5)		152 (26.4)	417 (23.5)	
**Education level**				< 0.001			0.008
None	1908 (39.9)	204 (37.6)	928 (48.7)		215 (37.5)	561 (31.9)	
Primary school	1696 (35.5)	221 (40.7)	677 (35.5)		207 (36.1)	591 (33.6)	
Technical school	363 (7.6)	33 (6.0)	92 (4.8)		46 (8.0)	192 (10.9)	
Secondary school	279 (5.8)	29 (5.3)	67 (3.5)		36 (6.2)	147 (8.3)	
University	531 (11.1)	55 (10.1)	141 (7.4)		69 (12.0)	266 (15.1)	
Unknown	35 (0.7)						
**BMI**				< 0.001			0.475
Normal weight	889 (18.6)	167 (30.5)	424 (22.11)		67 (11.6)	241 (13.6)	
Overweight	2401 (49.9)	214 (39.1)	826 (43.07)		341 (59.3)	1020 (34)	
Obese	1512 (31.4)	166 (30.3)	668 (34.83)		167 (29.0)	511 (28.8)	
**Smoking habit**				< 0.001			0.110
Never	2795 (58.1)	397 (72.5)	1584 (82.6)		205 (35.6)	609 (34.4)	
Former	903 (18.7)	51 (9.3)	136 (7.0)		156 (27.1)	560 (31.6)	
Smoker	1111 (23.1)	99 (18.1)	197 (10.2)		214 (37.2)	601 (33.9)	
Unknown	3 (0.1)						
**Physical activity**				< 0.001			0.389
Low	1077 (22.4)	54 (9.9)	100 (5.2)		210 (36.5)	713 (40.2)	
Medium	798 (16.6)	65 (11.9)	182 (8.5)		141 (24.5)	410 (23.1)	
High	1160 (24.1)	153 (28.0)	510 (26.6)		132 (23.0)	365 (20.6)	
Very high	1777 (8.54)	275 (50.3)	1126 (58.7)		92 (16.0)	284 (16.0)	
**Alcohol consumption**				0.085			0.177
None	1504 (31.3)	237 (43.3)	932 (48.5)		72 (12.5)	263 (14.8)	
Drinker	2329 (48.4)	272 (49.7)	874 (47.60)		280 (48.7)	903 (51.0)	
Heavy drinker	979 (20.3)	38 (6.9)	73 (3.81)		223 (38.8)	606 (34.2)	
**Menopausal status**				< 0.001			
Premenopausal	906 (36.7)	276 (50.5)	630 (32.8)				
Postmenopausal	1188 (48.2)	193 (35.3)	995 (51.9)				
Perimenopausal	218 (8.8)	51 (9.3)	167 (8.7)				
Surgical Postmenopausal	153 (6.2)	27 (4.9)	126 (6.6)				
**Number of pregnancies**				< 0.001			
0	253 (10.4)	67 (12.5)	186 (9.8)				
1–2	762 (31.4)	194 (36.2)	568 (30.)				
≥3	1414 (58.2)	275 (51.3)	1139 (60.2)				
Unknown	36 (1.5)						
**Breastfeeding**				0.010			
No	464 (19.1)	123 (22.9)	341 (18.0)				
Yes	1964 (80.9)	412 (77.1)	1551 (82.0)				
Unknown	37 (1.5)						
**Oral contraceptive ever**				< 0.001			
No	1645 (66.7)	329 (30.1)	1316 (68.6)				
Yes	819 (33.2)	218 (39.9)	601 (31.4)				
Unknown	1 (0.04)						
**Hormone replacement therapy**				0.491			
No	2071 (87.7)	473 (88.6)	1598 (87.5)				
Yes	290 (12.3)	61 (11.4)	229 (12.5)				
Unknown	104 (4.2)						

*Chi-square

### Breast cancer

Among breast cancer patients, there were more smokers (18.1% vs 10.3%) than in the sub-cohort. Regarding physical activity, the sub-cohort had more participants in the “very high” classification than the cases: 58.7% vs 50.3% respectively. Other significant differences were observed in education level, BMI, menopausal status, number of pregnancies, breastfeeding and oral contractive use comparing breast cancer cases and sub-cohort participants (Table 1).

Percentage of detection was similar between cases and sub-cohort, around 68% of the samples showed levels above LOD (Supplementary material, Table 1). BPA geometric mean was slightly higher in cases than in participants of the sub-cohort (1.12 ng/ml vs 1.10 ng/ml) (*p* = 0.754) (Supplementary material, Table 1). Breast cancer cases presented higher percentage of detection above LOD (75.7% vs 63.4%) and higher GM (1.51 ng/ml vs 0.90 ng/ml) than participants from the sub-cohort (*p* = 0.010) between smoker (Supplementary material, Table 1). We found significant differences in serum BPA concentrations in the participants with secondary school when comparing cancer cases and sub-cohort (*p* = 0.002). However, there were no significant differences when observing other variables, neither those concerning women reproducibility (Supplementary material, Table 1).

Cox regression analyses showed no statistically significant association between BPA levels and breast cancer incidence in our cohort in the developed models (Table 2). There were no significant association when analyzing linear BPA in model A (HR = 1.047; *p* = 0.200), neither in logarithmic model (Models B, Table 2). In the same way, no significant effect was observed when the exposure variable was categorized into tertiles (Models C, Table 2).

**Table 2:** Cox regression and risk of breast and prostate cancer

		Breast cancer					Prostate cancer				
		N	HR	SE	p	95% CI	N	HR	SE	p	95% CI
**Model A**	**BPA levels (for 5 ng/ml increase)**	2306	1.047	0.037	0.200	0.98–1.12	2328	0.989	0.036	0.749	0.92–1.06
**Model B**	**log**_**2**_**(BPA)**	2306	1.011	0.024	0.655	0.97–1.06	2328	1.035	0.021	0.093	0.99–1.08
**Model C**	**Categorized BPA (values in ng/ml)**										
	<LOD	705	1	–	–	–	658	1	–	–	–
	Tertile 1 [0.2–1.8)	562	0.820	0.123	0.185	0.61–1.10	534	1.404	0.208	0.022	1.05–1.88
	Tertile 2 [1.8–5.1)	556	0.875	0.132	0.376	0.65–1.18	540	1.365	0.200	0.034	1.02–1.82
	Tertile 3 [5.1–68.9]	483	1.127	0.169	0.425	0.84–1.51	596	1.305	0.193	0.072	0.98–1.74

Model A: linear BPA stratified by center and age group, and adjusted by age, education level, BMI, physical activity, smoking status, alcohol consumption (and menopause, n° of pregnancies, breastfeeding, oral contraceptives, HRT and the interaction between BMI and menopause for women)

Model B: log2BPA stratified by center and age group, and adjusted by age, education level, BMI, physical activity, smoking status, alcohol consumption (and menopause, n° of pregnancies, breastfeeding, oral contraceptives, HRT and the interaction between BMI and menopause for women)

Model C: categorized BPA (<LOD category plus tertiles based on measurable values in ng/dl) stratified by center and age group, and adjusted by age, education level, BMI, physical activity, smoking status, alcohol consumption (and menopause, n° of pregnancies, breastfeeding, oral contraceptives, HRT and the interaction between BMI and menopause for women)

LOD: limit of detection; CI: confidence interval

### Prostate cancer

There were no significant differences between prostate cancer patients and the sub-cohort concerning lifestyle variables (Table 1).

Serum BPA levels showed no significant differences in prostate cancer participants compared with the sub-cohort (1.33 ng/ml vs 1.29 ng/ml; *p* = 0.809) (Supplementary material, Table 2). Non-significant differences in BPA levels were observed between cases and sub-cohort according to sociodemographic and life style characteristics (Supplementary material, Table 2).

Cox regression models showed no significant association of BPA serum levels and prostate cancer risk in linear model (Table 2). However, the result of the adjusted logarithmic model showed a positive association between an increase in BPA levels with the risk of prostate cancer (HR = 1.035; *p* = 0.093). Moreover, when categorizing BPA in tertiles we observed an increased risk of prostate cancer in model C: a 40% increase in risk of prostate cancer for tertile 1 (HR = 1.40; *p* = 0.022), 37% increase for tertile 2 (HR = 1.37; *p* = 0.034) and 31% increase for tertile 3 (HR = 1.31; *p* = 0.072) was observed with respect to values bellow the limit of detection (p-trend = 0.069) (Table 2).

## Discussion

This study longitudinally explores the potential contribution of participants’ exposure to BPA at recruitment in the development of breast and prostate cancer, over a relatively large follow-up time in Spain.

In our study, 70% of the population had detectable BPA values and its concentrations were similar among cancer cases and sub-cohort participants which is comparable to an epidemiological study in Korea (*n* = 167) were no significant differences in blood BPA levels between breast cases and controls were found (*p* = 0.42) [46].

Our results show no association for BPA serum concentrations and risk of incident breast cancer despite BPA is considered an ED with carcinogenic potential [31–33]. These results are in consonance with the study of Aschengrau et al., which found no association between adult occupational exposure to BPA and breast cancer diagnosis, although the exposure measure was through questionnaires (*n* = 1000 participants) [36]. In the same way, a population-based case–control study also showed no association between the urinary BPA levels and risk of breast cancer in postmenopausal Polish women (*n* = 575) [37]. However, recent reviews conclude that the evidence of the potential impact of BPA on human development of chronic diseases is sufficiently robust to raise concerns about BPA being an important health problem [32, 47–49]. These reviews are mostly based on animal and in vitro studies, due to the limited epidemiological studies. Experimental modelling suggests that BPA increases breast cancer susceptibility [47]. Still, few epidemiological studies have linked BPA to breast cancer. In this regard, high concentrations of serum BPA correlated with elevated mammographic breast density, a marker of breast cancer risk, in a study of postmenopausal women from Wisconsin (*n* = 264) [50]. Mammographic breast density increased from 12 to 17% when serum BPA levels increased to 0.55 ng/ml). However, case-control studies present a controversial validity when the possible risk factor is a biomarker, since this is measured in cases when the disease is already present and therefore the time sequence necessary to impute causality is not absent.

Regarding prostate cancer, we found a significant non-linear association with risk of prostate cancer when we categorized serum BPA in tertiles, as individuals with serum BPA levels in the 1st and 2nd tertile showed a 40 and 37% increased risk of prostate cancer respectively. In this regard, a case control study conducted in men with prostate cancer (*n* = 60) showed a much higher concentration of BPA in the urine of those patients in comparison with the control group [51]. In another case-control study in Hong-Kong [52] showed a positive exposure-response relationship between a cumulative BPA exposure index and prostate cancer, with the greatest and significant risk in the high versus reference category (OR = 1.57, 95% CI: 1.01–2.44). This study, however, did not had any biological measurement of BPA, as the cumulative BPA exposure index was based on self-reported information of the habitual use of specific type of food or beverage container including what the container is made of, the frequency of use, the handling practice, the heating and years of usage.

On the other hand, the biological matrix used for biomonitoring plays an important role. Serum BPA concentrations can be relatively unstable, representing recent exposures [53, 54]. In this regard, the biological matrix most commonly used to determine BPA is urine [55]. Indeed, some authors acknowledge urine concentrations as the best biomarker of BPA exposure, since metabolites in blood can be several orders of magnitude lower than in urine and it can be indicative of a relatively longer exposure period in comparison to other matrices [53]. Some studies point out that, among the biomonitoring matrices, urine contains the highest BPA concentrations, followed by serum [56] which implies a greater capacity to detect levels of exposure and also an improved estimator of medium-term exposure. However, there is still scarce studies about the correlation between matrices [56, 57]. The different biological matrices for measuring BPA have had many interrogations and discussion. Serum BPA specifically measures unconjugated BPA, and studies measuring BPA in blood are scarce, due to more complex logistics than urine. Therefore, our matrix selection could be interpreted as well as a contribution of the study to the scarce evidence of BPA in serum.

There is ample evidence about BPA exposure and risk of hormone dependent cancer in animal and in vitro studies both for breast cancer [58–70] and prostate cancer [71–76]. Conversely, the scarce epidemiological evidence on BPA exposure and risk of breast or prostate cancer studies have overall yielded to differing results, most likely due to different experimental designs, timing of exposure, and uncontrolled or residual confounding factors, such as the route of the administration of BPA, its degradation time or low exposure doses. The low range of BPA concentrations (ng/ml) is a consequence of its fast metabolism and short half-life in human body [77]. Moreover, human exposure to many potential EDs can be confounded because most existing cohorts and epidemiological studies (as our study) were designed to measure the impact of a single chemical without accounting for the effects of mixtures [78]. The heterogeneity of the populations of the studies, the different ways of assessing the exposure and, and the biological matrix can have a big influence in the exposure level as well as the different ways of evaluating the disease. In this regard, our method may have advantages, such as accessibility to practically the entire study population, taking advantage of the great coverage of public clinical records. However, we could have perhaps a possible underestimation of cases, or at least that usually happens when we rely on clinical records. Thus, different methodologies can result in inconsistent outcomes. Additional larger epidemiological studies are needed to obtain sufficient evidence and to identify the degree to which there is an association between low-dose BPA exposure and breast and prostate cancer risk.

On the other hand, some possible mechanisms of action of BPA carcinogenicity could be genetic damages, epigenetic effects, endocrine disruption, oxidative stress and mitochondrial dysfunction and cell signaling [31, 32]. It has been shown that BPA can interact with estrogen receptors, behaving as agonist or antagonist through endocrine receptor dependent signaling pathways [79]. These actions can lead to diverse changes in estrogen-target organs including mammary gland [9]. BPA can also regulate the proliferation and migration of prostate cancer cells and induce DNA adducts in prostate cancer cells [80–82]. Consequently, BPA plays a role in the pathogenesis of several endocrine disorders including female and male infertility, precocious puberty and hormone dependent tumors such as breast and prostate cancer [83]. Studies conducted in vitro have shown that the exposure of the human breast cancer cell line to BPA increased its proliferation and caused increased oxidative stress [84, 85]. Growing evidence suggests that BPA-induced damage is associated with oxidative stress [32, 86, 87] as BPA can disturb oxidative homeostasis through direct or indirect pathways, including cancer, infertility, and neurodegenerative diseases [88, 89]. In the epidemiological study of Yang et el., authors reported that BPA exposure apparently promotes oxidative stress and inflammation in women [90]. Regarding evidence for epigenetic alterations, several animal studies have identified plausible mechanisms of action of BPA on prostate cancer risk as early life BPA exposure provides a potential mechanism of action for low dose BPA [91, 92]. These mechanisms could induce the tumors of interest, which are hormone dependent as the constant induction of estrogen / androgen receptors, even at low doses, compared to endogenous hormones, could activate cell proliferation or inhibit protective mechanisms [93].

Our study has some limitations since the exposure to BPA was estimated by using serum concentrations at recruitment, and we do not have information on changes in BPA concentrations and covariates during the follow-up time as BPA may vary over time in our longitudinal design. In addition, the use of one BPA point measurement might not take into account intra-person and intra-day fluctuations, which might be relevant in certain populations [94, 95]. However, The use of one-spot samples, have been shown to be a reflection of BPA exposure in the population in some studies [96]. Though, BPA levels in the organism are not stable, as levels are higher the moment the person has been exposed and until BPA is metabolized and excreted 7 or 8 h after its incorporation into the body. BPA is rapidly conjugated and excreted by humans due to the efficient glucuronidation of BPA [77]. Moreover, we have to take into account that our population have been exposed to other pollutants. Therefore, possible associations from one single contaminant may be due to other highly correlated (and unmeasured) co-exposures, potentially including both persistent and non-persistent pollutants, or even a result of interactions among different co-exposures [97, 98]. Finally, although we adjusted for n° of pregnancies, breastfeeding, oral contraceptives, HRT and the interaction between BMI and menopause for women, we cannot exclude a potential residual confounding. Therefore, more research considering subclinical disease markers would shed light on the causality of the observed associations.

This study also has a number of strengths. The population considered is large and well representative of exposure to BPA in the 1990s. This characteristic is valuable, enabling us to study possible associations between BPA exposure at recruitment and certain chronic illnesses currently present in the participants. In this respect, it is of notable importance that 70% of the study population had detectable levels of BPA. We used previously-validated questionnaires which allow to have a precise characterization of the covariates. Besides, BPA was screened using validated analytical methodologies [43].

## Conclusions

We evidenced a similar percentage of detection of BPA among breast cancer cases and sub-cohort from our population, and slightly higher percentage of detection in prostate cancer participants than in the sub-cohort. We observed an increased risk of prostate cancer in the 1st and 2nd tertile of serum BPA. However, we found no associations between serum BPA concentrations and risk of breast cancer. Further investigation is needed to elucidate the potential influence BPA exposure on breast and prostate cancer risk.

## Supplementary Information

**Additional file 1: Table S1.** Serum BPA levels (ng/ml) (percentage above the limit of detection (LOD) and geometric mean (GM) with 95% confidence interval) by sociodemographic and life style characteristics in breast cancer cases and sub-cohort. **Table S2.** Serum BPA levels (ng/ml) (percentage above the limit of detection (LOD) and geometric mean (GM) with 95% confidence interval) by sociodemographic and life style characteristics in prostate cancer cases and sub-cohort.
