# Supplementary material for: Bisphenol-A exposure and risk of breast and prostate cancer in the Spanish European Prospective Investigation into Cancer and Nutrition study
Source: Environ Health. 2021 Aug 16;20:88. doi: 10.1186/s12940-021-00779-y (PMC8369702; doi:10.1186/s12940-021-00779-y)
Supplement: Supplementary file 1 — Additional file 1: Table S1. Serum BPA levels (ng/ml) (percentage above the limit of detection (LOD) and geometric mean (GM) with 95% confidence interval) by sociodemographic and life style characteristics in breast cancer cases and sub-cohort. Table S2. Serum BPA levels (ng/ml) (percentage above the limit of detection (LOD) and geometric mean (GM) with 95% confidence interval) by sociodemographic and life style characteristics in prostate cancer cases and sub-cohort. [file 12940_2021_779_MOESM1_ESM.docx]

**Supplementary material**

**Table 1. Serum BPA levels (ng/ml) (percentage above the limit of detection (LOD) and geometric mean (GM) with 95% confidence interval) by sociodemographic and life style characteristics in breast cancer cases and sub-cohort.**

|  | **Breast cancer cases** | | | **Sub-cohort** | | |  |
| --- | --- | --- | --- | --- | --- | --- | --- |
|  | **> LOD (%)** | **GM (ng/ml)** | **95% CI** | **> LOD (%)** | **GM (ng/ml)** | **95% CI** | **p*** |
| **Total** | 67.6 | 1.12 | 0.97-1.30 | 69.3 | 1.10 | 1.02-1.19 | 0.754 |
| **Center** |  |  |  |  |  |  |  |
| Gipuzkoa | 54.4 | 0.61 | 0.46-0.81 | 57.1 | 0.64 | 0.55-0.74 | 0.775 |
| Granada | 76.7 | 1.36 | 1.01-1.83 | 77.8 | 1.33 | 1.16-1.52 | 0.850 |
| Murcia | 65.6 | 1.01 | 0.77-1.32 | 71.5 | 1.18 | 1.02-1.36 | 0.275 |
| Navarra | 73.5 | 1.79 | 1.33-2.40 | 69.9 | 1.45 | 1.23-1.72 | 0.161 |
| **Age group** |  |  |  |  |  |  |  |
| <45 | 67.2 | 1.18 | 0.91-1.53 | 70.8 | 1.06 | 0.90-1.25 | 0.369 |
| 45-49 | 69.6 | 1.21 | 0.89-1.64 | 67.3 | 1.08 | 0.89-1.32 | 0.566 |
| 50-54 | 66.6 | 1.05 | 0.73-1.51 | 70.4 | 1.23 | 1.03-1.46 | 0.419 |
| 55-59 | 62.2 | 0.91 | 0.63-1.33 | 68.0 | 1.13 | 0.95-1.35 | 0.350 |
| 60+ | 72.8 | 1.22 | 0.81-1.86 | 69.4 | 1.03 | 0.88-1.19 | 0.417 |
| **Educational level** |  |  |  |  |  |  |  |
| None | 68.6 | 1.10 | 0.87-1.40 | 71.4 | 1.20 | 1.08-1.34 | 0.557 |
| Primary school | 67.4 | 1.09 | 0.86-1.37 | 68.2 | 1.13 | 0.99-1.29 | 0.796 |
| Technical school | 63.6 | 1.03 | 0.55-1.91 | 67.4 | 0.86 | 0.62-1.20 | 0.583 |
| Secondary school | 79.3 | 2.27 | 1.19-4.30 | 67.1 | 0.78 | 0.53-1.14 | 0.002 |
| University | 65.4 | 1.15 | 0.69-1.90 | 63.1 | 0.78 | 0.60-1.02 | 0.173 |
| **BMI** |  |  |  |  |  |  |  |
| Normal weight | 63.4 | 1.04 | 0.80-1.37 | 68.6 | 1.06 | 0.90-1.24 | 0.986 |
| Overweight | 74.3 | 1.40 | 1.11-1.77 | 69.5 | 1.10 | 0.98-1.24 | 0.070 |
| Obese | 63.2 | 0.91 | 0.70-1.18 | 69.4 | 1.13 | 0.99-1.29 | 0.171 |
| **Smoking habit** |  |  |  |  |  |  |  |
| Never | 67.2 | 1.07 | 0.90-1.28 | 70.3 | 1.15 | 1.05-1.25 | 0.552 |
| Former | 54.9 | 0.89 | 0.52-1.51 | 65.4 | 0.92 | 0.69-1.22 | 0.816 |
| Smoker | 75.7 | 1.51 | 1.08-2.10 | 63.4 | 0.90 | 0.73-1.14 | 0.010 |
| **Physical activity** |  |  |  |  |  |  |  |
| Low | 59.3 | 0.917 | 0.55-1.50 | 72.0 | 1.08 | 0.79-1.45 | 0.769 |
| Medium | 72.3 | 1.59 | 1.01-2.51 | 62.1 | 0.79 | 0.92-1.01 | 0.006 |
| High | 69.3 | 1.16 | 0.89-1.52 | 68.2 | 1.03 | 0.89-1.20 | 0.421 |
| Very high | 67.3 | 1.06 | 0.86-1.30 | 70.7 | 1.20 | 1.08-1.32 | 0.280 |
| **Alcohol consumption** |  |  |  |  |  |  |  |
| None | 67.9 | 1.23 | 0.98-1.55 | 69.8 | 1.11 | 1.00-1.24 | 0.350 |
| Drinker | 69.5 | 1.11 | 0.91-1.35 | 69.3 | 1.11 | 1.00-1.25 | 0.958 |
| Heavy drinker | 52.6 | 0.70 | 0.37-1.29 | 64.3 | 0.92 | 0.97-1.25 | 0.340 |
| **Menopausal status** |  |  |  |  |  |  |  |
| Premenopausal | 68.5 | 1.21 | 0.98-1.48 | 69.7 | 1.09 | 0.96-1.24 | 0.380 |
| Postmenopausal | 63.7 | 0.95 | 0.74-1.22 | 64.4 | 1.12 | 1.01-1.25 | 0.222 |
| Perimenopausal | 66.7 | 1.04 | 0.64-1.68 | 65.3 | 0.94 | 0.73-1.22 | 0.780 |
| Surgical Postmenopausal | 88.9 | 2.11 | 1.25-3.55 | 71.4 | 1.21 | 0.90-1.62 | 0.137 |
| **Number of pregnancies** |  |  |  |  |  |  |  |
| 0 | 64.2 | 1.22 | 0.77-1.93 | 66.7 | 0.87 | 0.69-1.10 | 0.224 |
| 1-2 | 69.6 | 1.23 | 0.96-1.59 | 65.0 | 1.04 | 0.90-1.21 | 0.222 |
| ≥3 | 67.6 | 1.04 | 0.85-1.28 | 72.4 | 1.19 | 1.08-1.31 | 0.272 |
| **Breastfeeding** |  |  |  |  |  |  |  |
| No | 68.3 | 1.12 | 0.82-1.53 | 68.6 | 1.01 | 0.84-1.20 | 0.569 |
| Yes | 67.8 | 1.13 | 0.96-1.34 | 69.8 | 1.13 | 1.04-1.23 | 0.925 |
| **Oral contraceptive ever** |  |  |  |  |  |  |  |
| No | 69.3 | 1.15 | 0.95-1.38 | 68.4 | 1.09 | 0.99-1.19 | 0.558 |
| yes | 65.1 | 1.09 | 0.86-1.38 | 71.2 | 1.13 | 0.99-1.29 | 0.814 |
| **Hormone replacement therapy** |  |  |  |  |  |  |  |
| No | 67.0 | 1.12 | 0.95-1.31 | 69.36 | 1.10 | 1.02-120 | 0.827 |
| Yes | 72.1 | 1.13 | 0.75-1.68 | 67.7 | 1.00 | 0.80-1.24 | 0.603 |

*p-value from Mann-Whitney test

**Table 2. Serum BPA levels (ng/ml) (percentage above the limit of detection (LOD) and geometric mean (GM) with 95% confidence interval) by sociodemographic and life style characteristics in prostate cancer cases and sub-cohort.**

|  | **Prostate Cancer Cases** | | | **Sub-cohort** | | |  |
| --- | --- | --- | --- | --- | --- | --- | --- |
|  | **> LOD (%)** | **GM (ng/ml)** | **95% CI** | **> LOD (%)** | **GM (ng/ml)** | **95% CI** | **p*** |
| **Total** | 74.4 | 1.33 | 1.16-1.52 | 70.9 | 1.29 | 1.19-1.40 | 0.809 |
| **Center** |  |  |  |  |  |  |  |
| Gipuzkoa | 66.2 | 0.86 | 0.69-1.07 | 57.7 | 0.72 | 0.61-0.84 | 0.137 |
| Granada | 88.8 | 2.55 | 1.76-3.71 | 84.5 | 2.15 | 1.86-2.49 | 0.593 |
| Murcia | 68.6 | 1.05 | 0.77-1.43 | 67.8 | 1.04 | 0.89-1.22 | 0.976 |
| Navarra | 82.7 | 2.05 | 1.64-2.57 | 71.9 | 1.65 | 1.38-1.96 | 0.294 |
| **Age group** |  |  |  |  |  |  |  |
| <45 | 64.7 | 1.10 | 0.56-2.15 | 66.2 | 1.06 | 0.89-1.27 | 0.952 |
| 45-49 | 75.2 | 1.62 | 1.15-2.28 | 70.9 | 1.47 | 1.19-1.81 | 0.531 |
| 50-54 | 70.8 | 1.21 | 0.89-1.62 | 70.7 | 1.40 | 1.17-1.68 | 0.270 |
| 55-59 | 74.4 | 1.22 | 0.96-1.56 | 74.8 | 1.38 | 1.15-1.66 | 0.412 |
| 60+ | 78.9 | 1.44 | 1.12-1.85 | 72.1 | 1.25 | 1.06-1.47 | 0.334 |
| **Educational level** |  |  |  |  |  |  |  |
| None | 75.8 | 1.44 | 1.15-1.80 | 71.1 | 1.30 | 1.12-1.50 | 0.511 |
| Primary school | 73.4 | 1.33 | 1.05-1.67 | 70.9 | 1.32 | 1.15-1.52 | 0.956 |
| Technical school | 69.5 | 1.04 | 0.65-1.66 | 64.6 | 1.02 | 0.79-1.30 | 0.941 |
| Secondary school | 75.0 | 1.37 | 0.76-2.46 | 74.8 | 1.57 | 1.19-2.07 | 0.730 |
| University | 75.3 | 1.20 | 0.82-1.75 | 72.9 | 1.29 | 1.05-1.59 | 0.665 |
| **BMI** |  |  |  |  |  |  |  |
| Normal weight | 65.6 | 0.89 | 0.59-1.33 | 67.6 | 1.07 | 0.87-1.33 | 0.423 |
| Overweight | 75.3 | 1.37 | 1.15-1.63 | 71.1 | 1.30 | 1.17-1.45 | 0.596 |
| Obese | 76.0 | 1.46 | 1.14-1.88 | 72.2 | 1.40 | 1.20-1.63 | 0.945 |
| **Smoking habit** |  |  |  |  |  |  |  |
| Never | 72.2 | 1.19 | 0.95-1.50 | 71.7 | 1.41 | 1.23-1.62 | 0.261 |
| Former | 71.8 | 1.27 | 0.97-1.66 | 71.8 | 1.22 | 1.06-1.41 | 0.859 |
| Smoker | 78.5 | 1.52 | 1.23-1.89 | 69.2 | 1.25 | 1.09-1.44 | 0.184 |
| **Physical activity** |  |  |  |  |  |  |  |
| Low | 73.3 | 1.26 | 1.01-1.57 | 73.1 | 1.36 | 1.20-1.54 | 0.526 |
| Medium | 75.9 | 1.40 | 1.06-1.83 | 66.8 | 1.12 | 0.95-1.33 | 0.202 |
| High | 78.0 | 1.49 | 1.13-1.98 | 71.2 | 1.33 | 1.11-1.58 | 0.459 |
| Very high | 69.6 | 1.19 | 0.84-1.67 | 71.1 | 1.38 | 1.12-1.69 | 0.469 |
| **Alcohol consumption** |  |  |  |  |  |  |  |
| None | 75.0 | 1.35 | 0.911-2.02 | 75.3 | 1.59 | 1.29-1.97 | 0.446 |
| Drinker | 73.6 | 1.26 | 1.04-1.52 | 70.9 | 1.23 | 1.10-1.38 | 0.940 |
| Heavy drinker | 75.3 | 1.42 | 1.13-1.77 | 69.1 | 1.27 | 1.11-1.47 | 0.435 |

*p-value from Mann-Whitney test
